# The Association between Self-Reported Sleep Quality and Metabolic Syndrome

**DOI:** 10.1371/journal.pone.0054304

**Published:** 2013-01-14

**Authors:** Hao-Chang Hung, Yi-Ching Yang, Horng-Yih Ou, Jin-Shang Wu, Feng-Hwa Lu, Chih-Jen Chang

**Affiliations:** 1 Division of Endocrinology and Metabolism, Department of Internal Medicine, National Cheng Kung University Hospital, Tainan, Taiwan; 2 Department of Family Medicine, National Cheng Kung University Hospital, Tainan, Taiwan; 3 Department of Family Medicine, College of Medicine, National Cheng Kung University, Tainan, Taiwan; 4 Department of Internal Medicine, College of Medicine, National Cheng Kung University, Tainan, Taiwan; Azienda Policlinico S. Orsola-Malpighi, Italy

## Abstract

**Objectives:**

Short and long sleep duration are associated with metabolic syndrome. However, there is limited research on the association between sleep quality and metabolic syndrome, and thus the aim of this study is to investigate this relationship.

**Materials and Methods:**

The cross-sectional baseline data were collected from the decoded database of the Prevention Health Center of National Cheng Kung University Hospital from 2002 to 2006. The diagnosis of metabolic syndrome was according to the statement of the American Heart Association/National Heart, Lung, and Blood Institute. Sleep quality was assessed using the Pittsburgh Sleep Quality Index (PSQI). A higher global PSQI score indicates poorer sleep quality, and a global PSQI score greater than five differentiates poor from good sleepers.

**Results:**

Of the 3,435 subjects recruited, 899 (26.2%) had metabolic syndrome. Subjects with metabolic syndrome had higher PSQI and prevalence of poor sleepers than those without metabolic syndrome. The multivariate lineal regression analysis showed that female gender, metabolic syndrome, sleep duration, snoring, alcohol drinking, and habitual exercise were independent predictors of PSQI. When substituting metabolic syndrome with the five components, hyperglycemia and low high-density lipoprotein cholesterol (HDL-C) were positively associated with PSQI. The multivariate logistic regression analyses showed that female gender, metabolic syndrome, sleep duration, and snoring were independently associated with being poor sleepers. Of the five components, only low HDL-C was an independent predictor of being poor sleepers.

**Conclusions:**

Subjects with metabolic syndrome have higher global PSQI scores and a higher risk of being poor sleepers. Of the five components of metabolic syndrome, hyperglycemia and low HDL-C are independently associated with the global PSQI scores, while low HDL-C is an independent predictor of being poor sleepers.

## Introduction

Adequate sleep quality and quantity are important for the normal functioning of daily metabolic and hormonal processes and appetite regulation [1]. Chronic sleep debt, which is increasingly common in developed countries, is associated with metabolic and endocrine alterations that may have pathological consequences in the long term [2]. Furthermore, many studies have shown that sleep duration is associated with diabetes [3], [4], obesity [5], [6], cardiovascular disease [7], [8], and all-cause mortality [9]. In addition to sleep duration, reduced sleep quality without changes in duration is associated with insulin resistance [10], [11], and sleep disturbances are also related to diabetes [4] and all-cause mortality [12].

Metabolic syndrome represents a cluster of coronary heart disease risk factors, including obesity, elevated blood pressure, high fasting plasma glucose and triglyceride concentrations, and low serum high-density lipoprotein cholesterol levels [13]. It is associated with several potentially modifiable lifestyle factors, such as smoking, alcohol consumption, and physical inactivity [14]. Furthermore, both short and long sleep duration are associated with metabolic syndrome [15]–[17]. However, there is limited research on the association between sleep quality and metabolic syndrome and its components. Recently, in the Coronary Artery Risk Development in Young Adults Sleep Study, where actigraphy was used to assess sleep, both shorter sleep duration and greater sleep fragmentation were associated with higher body mass index [18], but not with markers of glucose metabolism in non-diabetic middle-aged adults [19].

The Pittsburgh Sleep Quality Index (PSQI) is a widely used and well-validated measure of sleep quality, and is suitable for epidemiologic investigations [20]. Jennings et al. showed that the PSQI is associated with metabolic syndrome in a sample of two hundred and ten Caucasian adults [21]. However, their sample size was relatively small, and they did not exclude conditions that might affect sleep quality, such as depression, thyroid disease and chronic pain. Therefore, the aim of this study is to investigate the association between the components of metabolic syndrome and sleep quality, using the PSQI.

## Materials and Methods

The cross-sectional baseline data were collected from the decoded database of the Prevention Health Center of National Cheng Kung University Hospital from 2002 to 2006. The study protocol was approved by the Institutional Review Board of the National Cheng Kung University Hospital. Subjects who received a health examination were included. All the subjects completed a structured questionnaire, which included demographic information, medical history, medication history, sleep assessment, smoking, alcohol drinking, and exercise habits. Informed consent was obtained from all of the participants. Because they only agreed to have their questionnaire data and related examination results analyzed anonymously, any identifying information was kept confidential. Those subjects who had the following conditions or diseases were excluded: 1) self-reported depression, anxiety, or other psychiatric disorders; 2) serum creatinine >132.6 µmol/l; 3) serum aspartate aminotransferase (AST) or alanine aminotransferase (ALT) levels more than twice the normal upper limit; 4) cancers; 5) history of obstructive sleep apnea; 6) history of thyroid diseases; 7) history of cerebrovascular diseases; and 8) chronic pain.

Sleep quality was measured using the Pittsburgh Sleep Quality Index (PSQI), which is a validated self-rated questionnaire that assesses sleep quality and disturbances over a one-month time interval [20]. The 19 items in the index generate seven component scores that reflect sleep problems in the areas of subjective sleep quality, sleep latency, sleep duration, habitual sleep efficiency, sleep disturbances, use of sleep medications, and daytime dysfunction. The sum of the scores for these seven components produces a global sleep quality score with a range of 0–21 points. A higher global PSQI score indicates poorer sleep quality, and a global PSQI score greater than 5 has a diagnostic sensitivity of 89.6% and specificity of 86.5% in differentiating poor from good sleepers. Snoring is defined as coughing or snoring loudly three times or more a week during the past month. The Chinese version of the Pittsburgh Sleep Quality Index has an overall reliability coefficient of 0.82–0.83, and acceptable test-retest reliability with a coefficient of 0.77–0.85 [22].

Wearing light indoor clothes, each subject's body height (to the nearest 0.1 cm), weight (to the nearest 0.1 kg), and waist circumference (to the nearest 0.1 cm) was measured. Waist circumference measurement was performed at the end of normal expiration in duplicate on bare skin midway between the lower rib margin and the iliac crest. Body mass index (BMI) was calculated as weight (in kilograms) divided by height (in meters) squared. For blood pressure measurement, each subject rested 10 minutes in a supine position in a quiet ambience, and measurements were obtained in a fasting state between 08∶00 and 10∶00 AM. Two blood pressure readings, separated by intervals of at least 5 min, were taken with an appropriate-sized cuff wrapped around the right upper arm using a DINAMAP vital sign monitor (model 1846SX; Critikon Inc., Irvine, CA).

All subjects underwent 12-h overnight fasting and blood sampling for basic biochemical examinations, including for creatinine, AST, ALT, total cholesterol, triglyceride, high-density lipoprotein cholesterol (HDL-C), and fasting plasma glucose (FPG). Blood glucose was measured by a hexokinase method (Roche Diagnostic GmbH, Mannheim, Germany) with an auto-analyzer Hitachi 747E. Serum total cholesterol, triglycerides, and HDL-C levels were determined in the central laboratory of National Cheng Kung University Hospital with the same autoanalyzer.

Metabolic syndrome was defined according to the statement of the American Heart Association/National Heart, Lung, and Blood Institute for Asian populations [13]. A subject was said to have metabolic syndrome when any three of the following five risk factors were present: 1) waist circumference (WC) ≥90 cm for men, ≥80 cm for women; 2) systolic blood pressure (SBP) ≥130 mmHg, or diastolic pressure (DBP) ≥85 mmHg, or current medication for hypertension; 3) HDL-C <1.03 mmol/l in men, <1.3 mmol/l in women; (4) fasting plasma glucose (FPG) ≥5.6 mmol/l or current use of antidiabetic therapy; and (5) triglyceride ≥1.7 mmol/l. Habitual exercise was defined as vigorous exercise three or more times per week when the subjects engaged in activities sufficient to work up a sweat [23]. Smoking habit was classified into smokers (defined as at least one pack/month for the previous six months) and non-smokers. Alcohol drinking habit was classified into drinkers (defined by at least one drink per week for the previous six months) and non-drinkers.

### Statistics

SPSS software (version 17.0; SPSS, Chicago, IL) was used for statistical analysis. All normally distributed continuous variables were expressed as means ± SD. Study subjects were categorized into two groups: with and without metabolic syndrome. Chi-square tests were used to analyze the difference in variables between groups. Multivariate linear regression analysis was conducted to identify independent predictors of the global PSQI score. Initially, the independent variables included age, gender, the presence of metabolic syndrome, short (<6 vs. 6–8 hours) and long (>8 vs. 6–8 hours) sleep duration. Further adjustments for snoring, alcohol drinking, smoking, habitual exercise, and creatinine were made. The same independent variables, except for the presence of metabolic syndrome, were then used in a multivariate linear regression analysis relating each component of the metabolic syndrome to the global PSQI score. Multivariate logistic regression analysis was conducted to identify independent determinants of poor sleepers (global PSQI score >5) by using the same variables stepwise in the multivariate linear regression analyses. A *p* value of less than 0.05 was considered statistically significant.

## Results

Of the total 3,435 subjects recruited, 899 (26.2%) had metabolic syndrome. Subjects with metabolic syndrome were older, and had higher BMI, WC, SBP, DBP, total cholesterol, triglyceride, and creatinine levels, and a higher prevalence of being elderly (aged ≥60 years), snoring, smoking habit, and alcohol drinking, but had lower HDL-C concentrations and less prevalence of being female or doing habitual exercise, than those without metabolic syndrome. In addition, the prevalence of central obesity, elevated blood pressure, hyperglycemia, hypertriglyceridemia, and low HDL-C was also significantly higher in subjects with metabolic syndrome (Table 1). Subjects with metabolic syndrome had higher global PSQI scores (6.7±3.2 vs. 6.1±2.4, p<0.001) and a greater prevalence of poor sleepers (63.4% vs. 53.5%, p<0.001) than those without metabolic syndrome.

**Table 1:** Comparisons of clinical characteristics between subjects with and without metabolic syndrome.

	Metabolic syndrome		P value
	Yes	No	
N	899 (26.2)	2536 (73.8)	–
Age (years)	50.8±11.8	43.5±11.8	<0.001
Age ≥60 years (%)	22.8	9.1	<0.001
Female gender (%)	26.8	38.9	<0.001
Global PSQI score	6.7±3.2	6.1±2.4	<0.001
Poor sleepers (%)	63.4	53.5	<0.001
BMI (kg/m^2^)	27.1±3.3	23.4±3.0	<0.001
Waist circumference (cm)	92.0±8.3	80.4±8.9	<0.001
SBP (mmHg)	139.5±16.9	120.5±16.0	<0.001
DBP (mmHg)	75.2±9.1	66.6±8.9	<0.001
FPG (mmol/l)	5.9±2.0	4.9±0.8	<0.001
Total cholesterol (mmol/l)	5.2±1.0	5.0±0.9	<0.001
Triglyceride (mmol/l)	2.3±1.4	1.2±0.6	<0.001
HDL-C (mmol/l)	1.0±0.2	1.3±0.3	<0.001
Creatinine (µmol/l)	81.4±15.9	77.5±15.6	<0.001
Smoking habit (%)	22.7	16.4	<0.001
Alcohol drinking (%)	21.1	16.9	<0.01
Habitual exercise (%)	36.0	40.0	<0.05
Central obesity (%)	79.3	19.2	<0.001
Elevated blood pressure (%)	73.6	23.0	<0.001
Hyperglycemia (%)	47.9	6.6	<0.001
Hypertriglyceridemia (%)	71.6	13.7	<0.001
Low HDL (%)	78.0	24.4	<0.001
Snoring ≥3 times/week (%)	33.9	17.6	<0.001

Data are expressed as means ± SD or %.

Abbreviations: SBP, systolic blood pressure; DBP, diastolic blood pressure; FPG, fasting plasma glucose. HDL cholesterol, high-density lipoprotein cholesterol.

The results of multivariate linear regression analysis of clinical variables and the global PSQI score in Table 2 show that female gender (p<0.001), metabolic syndrome (p<0.001), and short sleep duration (p<0.001) were positively associated with the global PSQI score after adjusting for age and gender (Model 1), whereas long sleep duration (p<0.001) was negatively associated with it. Further adjusting for snoring, habitual exercise, alcohol drinking, smoking habit, and creatinine (Model 2) showed that female gender (p<0.001), metabolic syndrome (p<0.001), short sleep duration (p<0.001), snoring (p<0.001), and alcohol drinking (p<0.05) were positively associated with the global PSQI score, whereas long sleep duration (p<0.001) and habitual exercise (p<0.05) were negatively associated with it. When substituting metabolic syndrome in Model 2 with the five components of metabolic syndrome (Model 3), hyperglycemia (p<0.05) and low HDL-C (p<0.005) were positively associated with the global PSQI scores.

**Table 2:** Multivariate linear regression analyses of clinical variables and the global PSQI score.

	Model 1	Model 2	Model 3
	Beta (95% CI)	Beta (95% CI)	Beta (95% CI)
Age ≥ 60 years, yes vs. no	0.196 (-0.071 ∼ 0.463)	0.195 (-0.072 ∼ 0.463)	0.227 (-0.052 ∼ 0.505)
Gender, female vs. male	0.703* (0.519 ∼ 0.886)	0.843* (0.566 ∼ 1.121)	0.824* (0.541 ∼ 1.107)
Metabolic syndrome, yes vs. no	0.960* (0.756 ∼ 1.163)	0.868* (0.662 ∼ 1.073)	—
Sleep duration (hours)			
< 6 vs. 6-8	1.025* (0.568 ∼ 1.483)	0.973* (0.515∼ 1.431)	1.018* (0.557 ∼ 1.480)
> 8 vs. 6-8	-0.520* (-0.702 ∼ -0.339)	-0.551* (-0.733 ∼ -0.370)	-0.557* (-0.740 ∼ -0.374)
Snoring ≥ 3 times/week, yes vs. no	—	0.477* (0.260 ∼ 0.694)	0.522* (0.303 ∼ 0.742)
Alcohol drinking, yes vs. no	—	0.254‡ (-0.012 ∼ 0.496)	0.264‡ (0.019 ∼ 0.510)
Smoking, yes vs. no	—	0.204 (-0.041 ∼ 0.450)	0.228 (-0.022 ∼ 0.477)
Habitual exercise, yes vs. no	—	-0.221‡ (-0.402 ∼ -0.040)	-0.238‡ (-0.421 ∼ -0.055)
Creatinine (µmol/l)	—	0.000 (-0.008 ∼ 0.008)	0.001 (-0.007 ∼ 0.009)
Central obesity, yes vs. no	—	—	0.130 (-0.072 ∼ 0.331)
Hyperglycemia, yes vs. no	—	—	0.296‡ (0.049 ∼ 0.543)
Elevated blood pressure, yes vs. no	—	—	0.143 (-0.059 ∼ 0.344)
Low HDL, yes vs. no	—	—	0.293† (0.097 ∼ 0.489)
Hypertriglyceridemia, yes vs. no	—	—	-0.061 (-0.280 ∼ 0.159)

Dependent variable: global PSQI score.

* P<0.001; †P<0.005; ‡ P<0.05.

The relationship between poor sleepers (global PSQI >5) and metabolic syndrome was examined with multivariate logistic regression analyses (Table 3). The results showed that female gender (p<0.001), metabolic syndrome (p<0.001), short sleep duration (p<0.005), long sleep duration (p<0.001), and snoring (p<0.005) were independently associated with being poor sleepers after adjusting for being elderly, alcohol drinking, smoking, habitual exercise, and creatinine (Model 1). Of the five components of metabolic syndrome (Model 2), only low HDL-C (p<0.05) was an independent predictor of being a poor sleeper.

**Table 3:** Multivariate logistic regression analyses of poor sleepers (global PSQI score >5) and metabolic syndrome.

	Model 1	Model 2
	OR (95% CI)	OR (95% CI)
Age ≥60 years, yes vs. no	1.061 (0.857∼1.313)	1.097 (0.881∼1.366)
Gender, female vs. male	1.588* (1.275∼1.978)	1.545* (1.238∼1.928)
Metabolic syndrome, yes vs. no	1.478* (1.254∼1.741)	–
Sleep duration (hours)		
<6 vs. 6–8	1.943† (1.292∼2.923)	1.995† (1.327∼2.999)
>8 vs. 6–8	0.657* (0.569∼0.757)	0.656* (0.569∼0.756)
Snoring ≥ 3 times/week, yes vs. no	1.302† (1.095∼1.548)	1.346† (1.131∼1.601)
Alcohol drinking, yes vs. no	1.135 (0.938∼1.373)	1.147 (0.947∼1.390)
Smoking, yes vs. no	1.060 (0.874∼1.286)	1.075 (0.885∼1.307)
Habitual exercise, yes vs. no	0.871 (0.755∼1.005)	0.865‡ (0.750∼0.997)
Creatinine (µmol/l)	1.000 (0.994∼1.007)	1.001 (0.995∼1.007)
Central obesity, yes vs. no	–	1.004 (0.857∼1.175)
Hyperglycemia, yes vs. no	–	1.139 (0.937∼1.383)
Elevated blood pressure, yes vs. no	–	1.034 (0.883∼1.210)
Low HDL, yes vs. no	–	1.176‡ (1.009∼1.372)
Hypertriglyceridemia, yes vs. no	–	0.904 (0.761∼1.074)

Dependent variable: poorer sleepers (global PSQI score >5).

* P<0.001; †P<0.005; ‡ P<0.05.

## Discussion

Our results show that subjects with metabolic syndrome have a significantly higher global PSQI score, and that the presence of metabolic syndrome is associated with a 0.9 increase in this. Jennings et al. showed that an increase in the global PSQI score of 2.6 points is associated with a 44% risk of having metabolic syndrome after adjusting for age and gender in Caucasian adults [21]. Although Jennings et al. found positive relationships between global PSQI scores and fasting plasma glucose, waist circumference, and BMI, they did not use the definitions of poorer sleepers and metabolic syndrome and its components applied in this work. However, our study extends their finding that subjects with metabolic syndrome had a 55.2% higher risk of being poor sleepers than those without, and hyperglycemia and low HDL-C were independently associated factors of the global PSQI score, and low HDL-C was the only independent predictor of being a poor sleeper.

The mechanism underlying the association between metabolic syndrome and sleep quality remains unclear. Hypothalamic-pituitary-adrenal (HPA) hyperactivity plays a role in the pathogenesis of the metabolic syndrome [24], and activation of the HPA axis can lead to sleeplessness [25]. In addition, several short term studies show that sleep fragmentation or restriction lead to insulin resistance [2], [10], [11], [26], which appears to play a key role in the pathophysiology of metabolic syndrome [27]. Indeed, chronic sleep debt may have modulatory effects on the glucose metabolism and promote the development of the metabolic syndrome, resulting in sleep disorders which in turn lead to poor sleep quality [28].

We found that hyperglycemia is an independent determinant of the global PSQI score, and this result is consistent with Jennings et al [21]. In addition, previous studies showed that poor sleep quality is also associated with poor glycemic control [19], [29] and the development of type 2 diabetes [4]. Although recent studies suggest that sleep restriction reduces insulin sensitivity [2], [10], [11], [26], there are bidirectional interactions between sleep and the glucose metabolism [28]. Moreover, we recently found that impaired glucose tolerance is associated with poor sleep quality, independent of cardiometabolic risk factors [30]. Low HDL-C, but not hypertriglyceridemia, is an independent predictor of the global PSQI score and poor sleepers in our analyses, while Jennings et al. found no association between HDL-C or triglyceride concentrations and global PSQI scores [21]. The mechanism responsible for the association between dyslipidemia and sleep quality remains unclear. However, sleep restriction is associated with increased cortisol and ghrelin levels, sympathetic response and reduced leptin levels [31], [32], which may promote the development of atherogenic lipid profiles [33].

We found no association between high blood pressure and the global PSQI score, consistent with Jennings et al. [21]. However, in patients with stage 1 hypertension (160 mmHg > SBP >140 mmHg, and 100 mmHg > DBP >90 mmHg), global PSQI scores were higher in non-dippers as compared with dippers [34]–[36]. Being poor sleepers was associated with a three-fold increased risk of being non-dippers [34], and the PSQI score is an independent determinant for non-dipping hypertension [35]. The decline of blood pressure at night was inversely related with the global PSQI score and the activation of the sympathetic nervous system [36]. However, the blood pressures of our study subjects were lower than those found in these three earlier studies, and this may have caused the negative results found in the current work. While central obesity is not associated with the global PSQI score and poor sleepers in our analyses, Jennings et al. showed that BMI, waist circumference, and fat percentage are all positively associated with the global PSQI score [21]. The different results may be due to the different ethnicities, sample sizes, and metabolic syndrome diagnostic criteria used in both works.

Our analyses show that short sleep duration increased the risk of poor sleep quality, but long sleep duration decreased it. Similarly, Bidulescu et al. found an inverse relationship between continuous sleep duration and global PSQI score [37], and previous studies showed that the sleep fragmentation score, a measurement of sleep quality obtained by actigraphy, is inversely related to sleep duration [18], [38]. Previous works also showed that long sleepers had a lower global PSQI scores among older Caucasian men [39], middle-aged African American [37], and older Chinese [40], consistent with our results. Patel et al. showed that there are no differences in sleep stage distribution and sleep fragmentation between subjects who report long (≥9 hours) versus normal (7–8 hours) sleep durations [39]. The reason why long sleepers had better sleep quality, as assessed by PSQI, remains unclear. Aeschbach et al. suggested that long sleepers (>9 hours) may have longer biological nights due to having different circadian rhythms, compared to short sleepers (<6 hours) [41], since the nocturnal intervals of high plasma melatonin levels, increasing cortisol levels, low body temperature, and increasing sleepiness are longer in the former than the latter [41].

In the current study, habitual exercise is negatively associated with global PSQI scores and poor sleepers, and this is consistent with Bidulescu et al., which found that physical activity is associated with better sleep quality, based on tertiles of the global PSQI score [37].

There are several limitations in the present study. First, since this work is cross-sectional in nature, the causal relationship between metabolic syndrome and sleep quality score could not be established. Second, we did not measure sleep by objective means, such as actigraphy and polysomnography, and subjects with undiagnosed obstructive sleep apnea (OSA) might not have been excluded. However, we adjusted for snoring, a common symptom of OSA, in the regression analyses, and the association between metabolic syndrome and poor sleep quality persisted. In addition, self-reports measure sleep in usual circumstances, and provide an overall sense of sleep rather than measuring on particular nights, as occurs using actigraphy or polysomnography. Furthermore, the PSQI is not related to objective measures of sleep, such as wrist actigraphy and polysomnography, in African Americans [42]. Third, we did not assess the level of psychological or socioeconomic stress, although the association between several self-reported stress measurements and the global PSQI score have been inconsistent in earlier research [42]. Finally, we did not measure hormones, which are involved in the association between sleep quality and metabolic syndrome, such as insulin resistance, sympathoadrenal activity, ghrelin and leptin [10], [11], [43].

In summary, we found that subjects with metabolic syndrome have significantly higher global PSQI scores and higher risk of being poor sleepers than those without metabolic syndrome. Furthermore, as for the impact of the components of metabolic syndrome on sleep quality, hyperglycemia and low HDL-C were independently associated factors of the global PSQI score, while low HDL-C was the only independent predictor of being a poor sleeper. Therefore, it is recommended that subjects with metabolic syndrome, especially with hyperglycemia and low HDL-C, should undergo screening with regard to sleep quality in order to aid the early detection of poor sleepers in clinical practice.
